# Case report: Combined transarterial and direct approaches for embolization of a large mandibular arteriovenous malformation

**DOI:** 10.4103/0971-3026.76044

**Published:** 2011

**Authors:** Chiramel George Koshy, Shyamkumar N Keshava, Vinu Moses, Sudipta Sen

**Affiliations:** Department of Radiology, Christian Medical College, Vellore, Tamil Nadu, India; 1 Department of Pediatric Surgery, Christian Medical College, Vellore, Tamil Nadu, India

**Keywords:** Arteriovenous malformation, embolization, mandible

## Abstract

Arteriovenous malformations (AVMs) that involve the mandible are difficult lesions to treat, with traditional options being surgery and embolization. This article describes a large mandibular AVM that was treated with embolization using transarterial as well as direct puncture approaches. Follow-up imaging showed thrombosis of the vascular spaces of the malformation. There were no complications. The patient is doing well and is on follow-up.

## Introduction

Arteriovenous malformations (AVMs) of the mandible are rare lesions that have a variety of presentations, including gingival bleeding, dental loosening, swelling, and life-threatening hemorrhage.[1] This article describes a large mandibular AVM that was embolized using both transarterial and direct puncture approaches.

## Case Report

**Figure 1:**
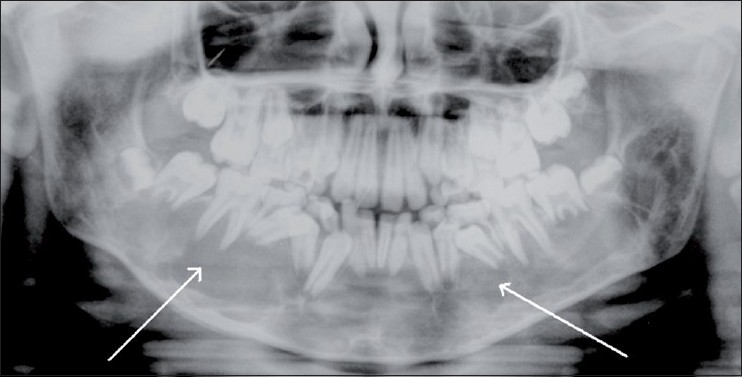
Orthopantomogram shows a large expansile osteolytic lesion (arrows) involving both sides of the body of the mandible

**Figure 2:**
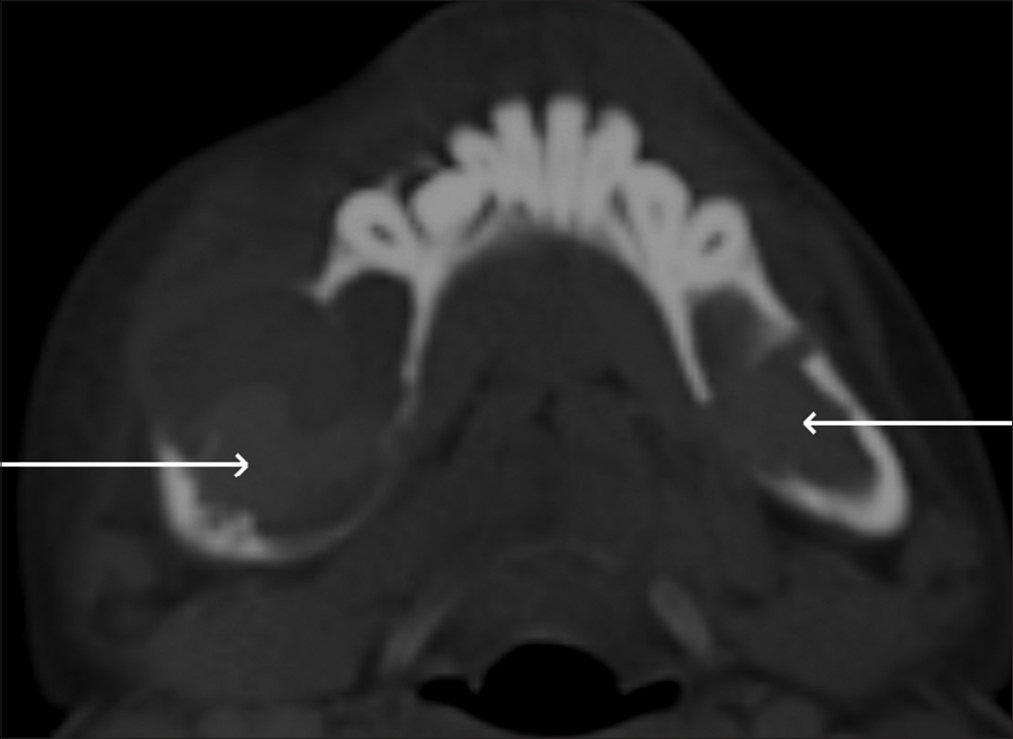
Axial, non-enhanced CT scan shows the expansile and osteolytic nature of the lesion, with hyperdense areas within (arrows) signifying partial thrombosis

**Figure 3 (A,B):**
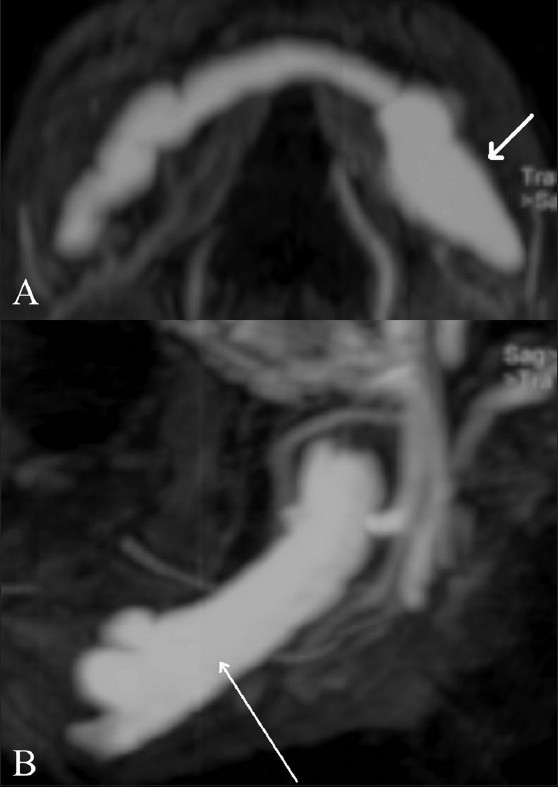
Axial (A) and sagittal (B) contrast enhanced T1W MR images show the arteriovenous malformation (AVM) (arrows) involving the whole of the body of the mandible

An 11-year-old boy presented with a few years’ history of gradual generalized enlargement of the mandible. An orthopantomogram [Figure 1] showed a large, well-defined, osteolytic lesion with sclerotic margins within the body of the mandible. A CT scan confirmed the presence of an expansile osteolytic lesion involving the body of the mandible [Figure 2], with enhancement of the lesion. The Doppler study revealed high-velocity blood flow within the vascular spaces of the lesion, suggesting the presence of an AVM. MRI studies were also carried out [Figure3A and B].

**Figure 4:**
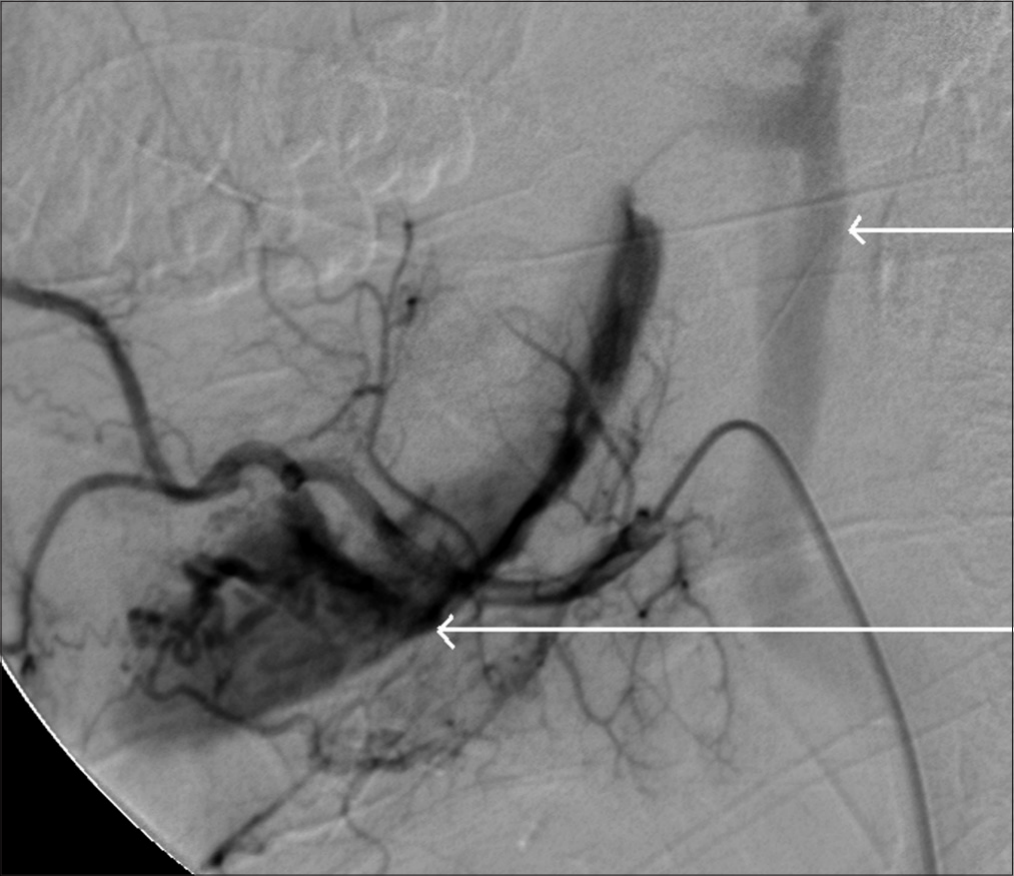
Lateral DSA image shows venous drainage of the AVM (long arrow) into the left internal jugular vein (short arrow)

**Figure 5:**
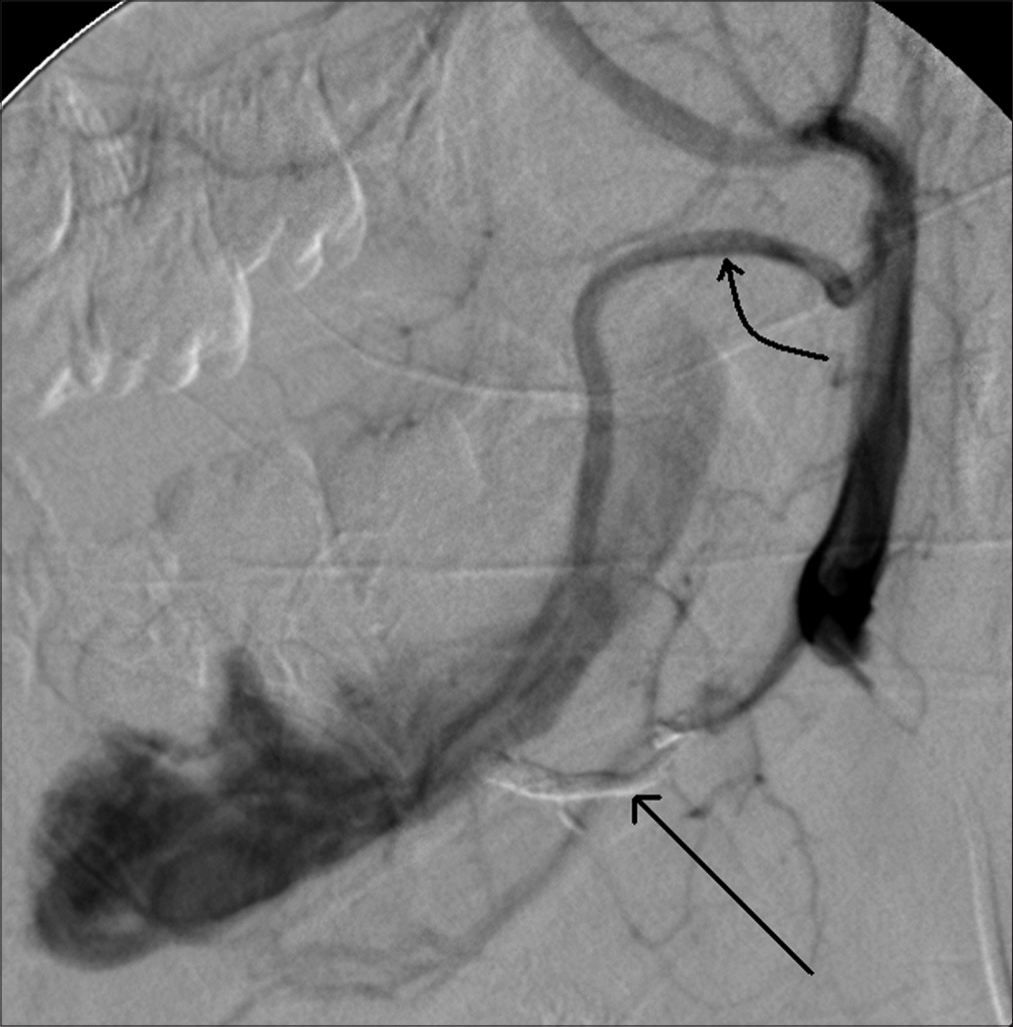
Lateral representative DSA image, of the left external carotid artery shows the embolized branch of the lingual artery seen as a glue-cast (straight arrow) and a hypertrophied anterior branch (curved arrow) from the terminal external carotid artery, supplying the AVM.

**Figure 6 (A,B):**
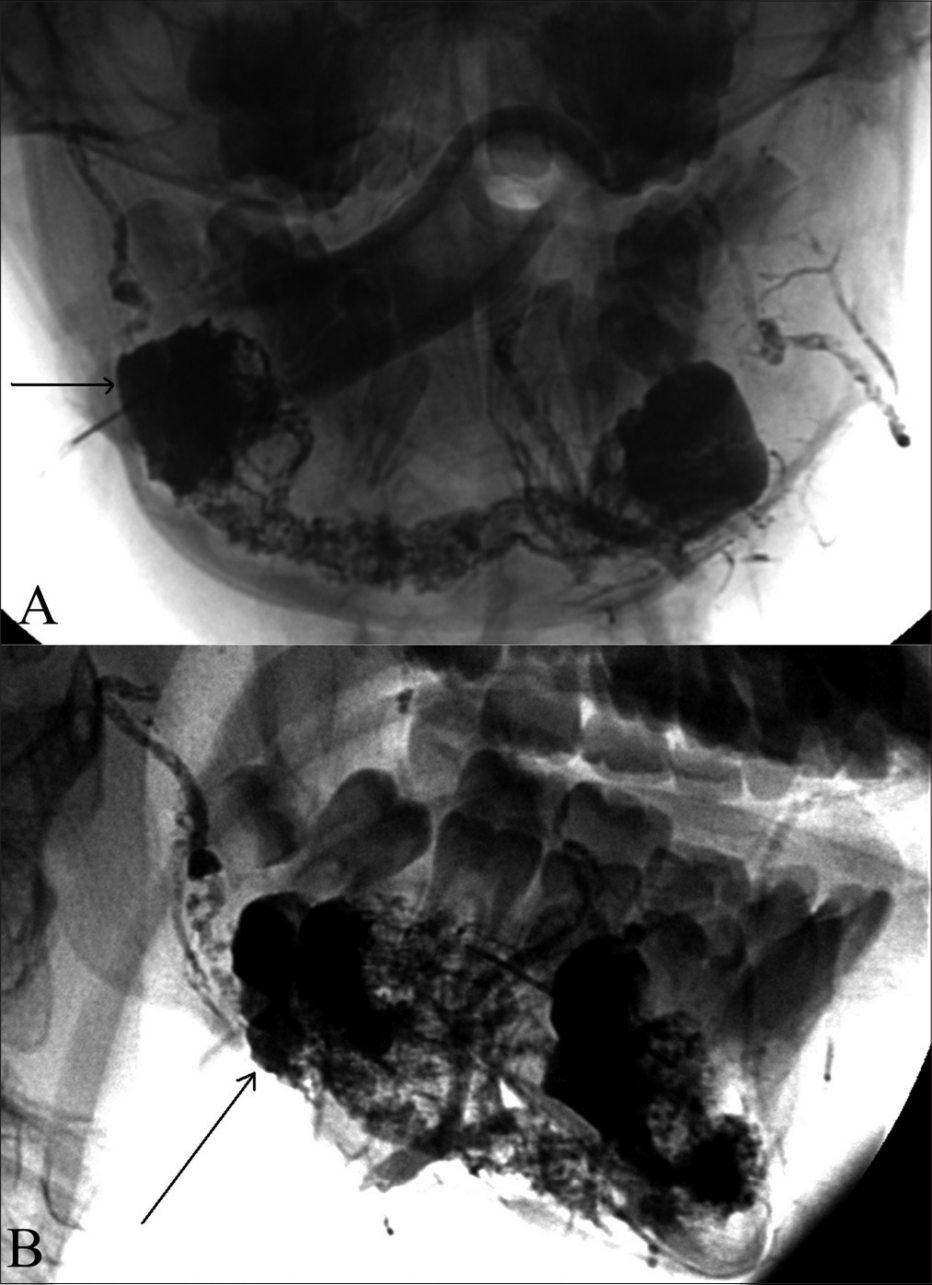
Frontal (A) and lateral (B) post-embolization radiographs after direct percutaneous glue injection show complete filling of the AVM (arrow) in the mandible with a mixture of N-butyl cyanoacrylate glue and radioopaque contrast agent (Lipiodol)

Digital subtraction angiography demonstrated a large AVM within the body of the mandible supplied by branches of both external carotid arteries and draining via a large intraosseous venous sac into the left internal jugular vein [Figure 4]. The feeding arteries were superselectively cannulated and 33% N-butyl cyanoacrylate glue (Histoacryl, B. Braun, Germany) was injected [Figure 5]. Doppler evaluation could demonstrate a reduction in the blood flow into the venous sac. The venous sac was then directly punctured under USG guidance through a defect in the bone using a 22G needle. Three milliliters of 20% Histoacryl glue was injected through this needle using fluoroscopic guidance [Figure 6].

**Figure 7 (A,B):**
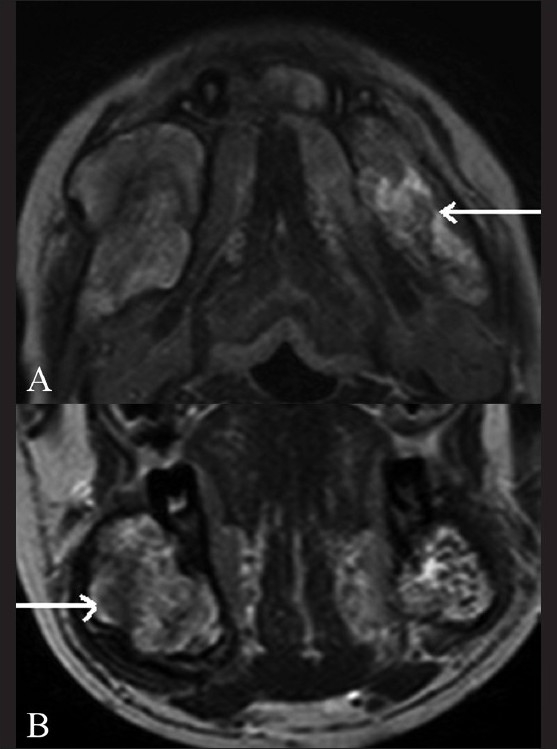
Post-embolisation T2W axial (A) and coronal (B) MRI images show thrombosis (arrow) of the AVM in the mandible and the absence of flow voids

The patient did well after the embolization procedure and had no complications. An MRI scan of the mandible performed a few days later showed thrombosis within the vascular spaces of the AVM [Figure 7]. The patient has been asymptomatic since then and is on follow-up.

## Discussion

Mandibular AVMs are rare high-flow vascular malformations that can present with mandibular enlargement, asymmetry, pain, increased mobility of the teeth and hemorrhage that can be life-threatening.[1] It is difficult to treat these lesions, the options being surgery and embolization. Complete or partial resection of the mandible after transarterial embolization has been suggested,[2] but surgery could potentially result in malocclusion or disfiguration.[3]

Embolization of the nidus of the AVM using polyvinyl alcohol particles or N-butyl cyanoacrylate can result in a permanent cure, although treatment failures secondary to recanalization and rebleeding from collaterals have been described.[4–6] Embolization of the proximal segments of the feeding arteries should be avoided as the lesion can gradually recruit further arterial feeders from the internal carotid and vertebral arteries. Furthermore, this restricts access for later sessions of transarterial embolization.

Direct puncture of the nidus or the draining varix followed by embolization with N-butyl cyanoacrylate or coils has been described.[5,7–9] Although direct puncture of the venous varix could provide a simple access, care should be taken to avoid radiation to the operator’s hands. Embolization of the venous varix without first embolizing the arterial feeders could potentially raise the intranidal pressure and lead to hemorrhage. Transvenous embolization of the venous varix using the femoral vein route has been described.[3]

MRI can be used for follow-up imaging due to its ability to detect flow voids, thrombus, and reossification.[3] MRI was used instead of an angiogram for follow-up due to concerns regarding the additional radiation dose.

## Conclusion

Intraosseous AVMs are rare lesions that can be treated using endovascular methods on a case-to-case basis. In the treatment of a mandibular AVM, a combined approach with transarterial followed by venous embolization using a direct puncture of the venous sac is an effective strategy.
